# "The Uric Acid Fetish": The Decline of Its Flesh-Creeping Power

**Published:** 1915-08-28

**Authors:** 


### "THE URIC ACID FETISH."

### The Decline of its Flesh-creeping Power.

The fate of uric acid has been in many respects an unhappy one. Physiologists have brushed it aside as mere waste matter, while physicians, or at least some of them, have proclaimed it as the root of all evil. This latter creed has caught the popular fancy, and public alarm has only been quieted by the reiterated assurance that all danger can be banished by the systematic use of an effervescing tablet. Trading in the same direction, amateur dietetic "specialists" have erected their stalls and have vaunted their books and their schemes, not, we imagine, wholly without profit. The labourer, admittedly, is worthy of his hire. Nevertheless signs are not wanting that the thing has been overdone and that the public flesh cannot be made to creep so readily as of yore. Such serious rivals as blood-pressure, intestinal stasis, and toxæmia have entered the field, and the pride of place formerly allotted to uric acid is seriously challenged. The wisdom of the market-place adapts itself to the new situation, and we now learn that the dread acid is, after all, not really so black as it was painted.

One of the most impressive proofs of the conclusion just stated is found in the conversion of Mr. Eustace Miles, M.A. Of course he is an authority on diet is Mr. Eustace Miles, M.A. He has had the "extraordinary advantage of watching the results of the diet (or, rather, the many diets) at the Eustace Miles Restaurant." Still more, there falls on him "the need to taste various foods" at this elegant and philanthropic institution. Again, history includes an occasion on which he suffered from cramp after taking some sauce which he had innocently believed to be free from meat-juices; tomato soup made with meat-stock has proved equally disastrous; and an "extract," though arriving through the guileless medium of a publisher, provided a third catastrophe. In view of this last experience it is not surprising to find that his most recent book is published by "Eustace Miles, M.A." The society of the non-dietetic publisher, at least when armed with "extracts," is evidently a risky business. Accepting all these experiences as a guarantee of authority, we learn that, whereas once upon a time Mr. Miles was quite convinced that "uric acid was almost the only cause of many physical and of many mental troubles," his position now is "quite different." The bad eminence occupied by uric acid must be shared with "carbohydrate over-acidity," with "chromogens and crystals," and with the "poisons from wrong thoughts and wrong emotions." The change, we repeat, is an impressive one, and seems to justify the suspicion that the dog has had his day.

It is of interest to inquire how the conversion of Mr. Miles has been effected. The principal agent in securing this triumph, it appears, has been Mr. C. H. Collins. This gentleman, like Mr. Miles himself, has his credentials and experiences. To begin at the beginning, he has a father who has "done drawings for some of the leading authors." Then he himself "has used the microscope from an early age," and with it he is "now *au fait*." Still, among his general accomplishments, we learn that he is "a keen student of various philosophies," and Mr. Miles has been "struck" by his "power of putting two and two together." To come to his more technical equipment, Mr. Collins "has been through the orthodox training in the examination of the fixed blood and so forth" . . . and has been observed to "take many hours over a single case." In short, he is a "leading Clinical Analytical Expert."

So much for the man, and now for the methods which have brought Mr. Miles a captive to his triumphal car. They are summed up in a phrase—"The threefold examination," and the price is two guineas. First of all Mr. Collins—note the advantage of early training—actually examines the blood under "a high magnifying power" and watches the changes which take place as it "gradually dies." The father who has "done drawings" in his day must be proud of him. When the "dying" is over the clinical analytical expert has already information amply sufficient to initiate treatment, and the rest of his threefold system he pursues in the laboratory, where, to quote a single instance, he has been able to discover how "the neighbourhood of a powerful electric supply station must have affected the health." Is it any wonder that Mr. Eustace Miles was converted and that he resolved, together with Mr. C. H. Collins, to write a new book in which the resources of the restaurant should be blended with the threefold examination, to the confusion of uric acid and the relief of mankind? The name of the book stands at the head of this column, and, in view of what the authors say about themselves and one another, even the most guileless of our readers will have little difficulty in guessing its purpose. It is sufficient to add that provision is made for all purses. The full combination, an hour's interview with Mr. Eustace Miles and the threefold business by Mr. C. H. Collins, costs four guineas, but a single lesson on "liver and kidney troubles, etc.," can be had for the modest sum of two half-crowns; and between these extremes are intermediate stations where the more or less extravagant can find such opportunities as water treatment and mental helps at one guinea, or ten lessons in the cooking school for thirty shillings. Then, as what are elegantly called in commercial circles "side lines," there are "mind-building" lessons at three guineas for four and "essay and article writing and speaking" experiences at three guineas for five. The whole display announces the happy union of "real science" with the business instinct, and as a guarantee of our capacity to appreciate this we assure Mr. Eustace Miles that, whilst we may not, as he says, be qualified like Mr. C. H. Collins "to examine the living blood," we are capable of at least one of this gentleman's exploits—we can put "two and two together."

